# Diverse Pollination Networks Key to Ecosystem Sustainability

**DOI:** 10.1371/journal.pbio.0040012

**Published:** 2005-12-13

As animal extinctions continue at the rate of one every 16 years, it's unclear how declining biodiversity will disturb ecosystem dynamics. Of special concern are the pollinators, essential players in the reproductive biology of plants, the earth's primary producers. Millions of years of evolutionary coadaptations lie behind the perfect pairing of pollinator proboscis anatomy with plant flower structure, as well as the mechanisms plants use to attract reproductive assistants to their food rewards. Agave plants emit musky aromas that attract lesser long-nosed bats to nectar stores within their flowers, for example. As the bats travel from flower to flower, pollen collects and then falls from their fur, facilitating cross-pollination.

These mutually beneficial relationships are sometimes so specialized that the loss of one species threatens the existence of the other, raising troubling questions about the likely consequences of declining diversity in pollination networks. In a new study, Colin Fontaine et al. tackled this question by experimentally manipulating plant and pollinator interactions under natural conditions. The authors found strong functional relationships between different pollinators and plant communities, with the highest plant community sustainability associated with the most diverse group of pollinators. These findings suggest that loss of biodiversity in pollination networks may threaten the persistence of plant communities.

For their study, the authors chose plants with easy and harder access to food rewards—three open-flower and three tubular-flower species—and insects with short and longer mouthparts—three syrphid fly and three bumblebee species. In the spring of 2003, Fontaine et al. set up 36 plant communities in nylon-mesh enclosures in a meadow 80 kilometers (about 50 miles) southwest of Paris, after sterilizing the soil to destroy seeds and pathogens. They planted 30 adult plants in each plot at the same density, and then captured and released local pollinators into the cages during the flowering season (June–July 2003, 2004). To test all the possible plant–pollinator combinations, the authors set up three plant treatments (open flowers, tubular flowers, and both flower types), then applied three pollination treatments (flies, bees, and both insects) to each plant treatment.

A month after the first pollination treatments, the authors tallied all the fruit on each plant, then randomly selected five fruits per plant (excepting one plant species from each group that would have required harvesting the fruit) to estimate seed production per plant. During the seedling season, they totaled the plants and the seedlings to measure plant population and reproductive success. Pollinator identity determined fruit production, with bee-pollinated plants most productive, and the different plant groups responded differently to the two pollinator groups. As expected, short-mouthed syrphid flies pollinated only open flowers, while bees pollinated both plant types. As a result, tubular flowers produced far fewer fruits with syrphid pollinators while open-flower fruit production remained the same regardless of pollinator. Fruit production increased along with both plant and pollinator diversity. Seed production was a bit more complicated. Though bee-pollinated open flowers produced fewer seeds per plant than those pollinated by syrphids, higher fruit production compensated by producing more seedlings. Fruit production increased with pollinator diversity. [Fig pbio-0040012-g001]

**Figure pbio-0040012-g001:**
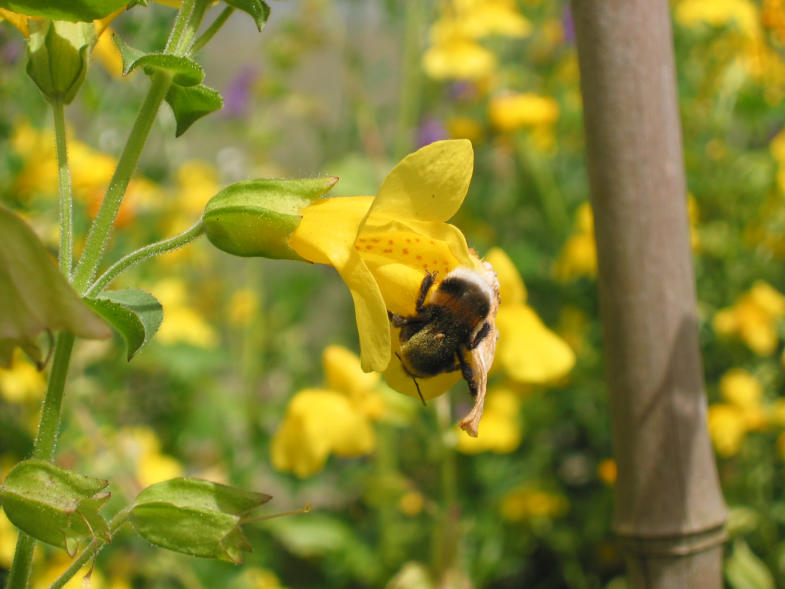
Loss of biodiversity in pollination networks may threaten the persistence of plant communities

As for long-term effects on plant reproductive capacity and success, tubular plant communities had fewer plants at the seedling stage than openflowered plants (and even fewer when pollinated by syrphids). The plant species number and total plant number increased when both pollinator groups were present, and were highest with maximum plant and pollinator diversity. Seedling production showed a similar pattern: mixed plant communities treated with both pollinators yielded the most seeds.

What happened? Not surprisingly, the pollinators stuck to their preferred plant: syrphids visited mostly open flowers, and bees visited mostly tubular flowers. Bees can pollinate open flowers but prefer tubular flowers when they have the choice, suggesting that bees may not fill a void left by a different pollinator. The presence of both pollinators allowed more appropriate pairings between insects and flowers—each performing a complementary role—leading to increased pollination efficiency and plant reproductive success.

While the study offers an admittedly pared down view of pollination networks, it demonstrates the value of studying the functional effects of pollination networks in the field. These results show that losing a species affects plant–pollinator communities, and that such losses may ultimately trigger further reductions in biodiversity, possibly reverberating through the food chain. With as many as 70% of plant species dependent on animal pollinators and at least 82 mammalian pollinator species and 103 bird pollinator species considered threatened or extinct, this is sobering news. —*Liza Gross*

